# “As If Neck Injuries Did Not Exist”: An Interview Study of Patients’ and Relatives’ Perceptions of Web Information on and Management of Whiplash Injuries in Sweden

**DOI:** 10.2196/ijmr.9881

**Published:** 2019-05-21

**Authors:** Gabriella Bernhoff, Christos Saripanidis, Bo Christer Bertilson

**Affiliations:** 1 Division of Family Medicine and Primary Care Department of Neurobiology, Care Sciences and Society (NVS) Karolinska Institutet Stockholm Sweden; 2 Academic Primary Healthcare Centre Stockholm County Council Stockholm Sweden

**Keywords:** self care, patient participation, health communication, patient portals, patient education handout, patient satisfaction, whiplash injuries, neck pain, chronic pain

## Abstract

**Background:**

If purposefully designed, patient information can help individuals make well-founded health care decisions. This study was initiated to improve the information on whiplash injuries found in the national health care portal Healthcare Guide 1177, operated by the Swedish government.

**Objective:**

The objective of this study was to describe the thoughts of patients and relatives on (1) information about whiplash injuries presented in the portal and (2) the Swedish health care system’s management of whiplash injuries.

**Methods:**

A total of 5 interviews were conducted with patients (n=10) who had experienced a whiplash injury and with relatives (n=3) of such patients. The interviews were taped, transcribed verbatim, and analyzed by means of conventional content analysis.

**Results:**

The following two themes emerged from the latent content analysis: (1) confidence and trust in the public health care system and (2) a disappointment with health care encounters.

**Conclusions:**

We found that most of the study participants felt distress due to insufficient information; respondents perceived a discrepancy between the public health care system's authority and the information provided. The Web information on whiplash injuries may greatly impact patients' care decisions as well as their physical, mental, and social well-being. We would recommend detailed patient information on whiplash injuries, with less emphasis on psychology and more data on pathophysiology, prognosis, and treatment.

## Introduction

### Background

Whiplash injury is an umbrella term for a type of trauma involving a sudden distortion of the neck, or an acceleration-deceleration injury. The injury mechanism is a forward and upward movement of the chest or the torso with great force, which creates a compression of the lower cervical spine. Simultaneously, there is an unphysiological so-called double curvature of the neck where the lower segments are in hyperextension and the upper joints are in flexion. The neck and head then move into extension, after which the head is thrown forward. Whiplash trauma is most common in traffic collisions [1] and also frequently occurs in sports, which accounts for approximately 10% of all neck injuries [2] (eg, ice hockey [3] or other contact sports [4]). The incidence of whiplash injury is estimated to be 3 out of 1000 inhabitants per year in Sweden [5], Western Europe, and North America [1]. A whiplash trauma can cause several different injuries. Symptoms include head and neck pain, radiating symptoms [6], and a multitude of other symptoms often referred to as whiplash-associated disorders (WADs). About half of the patients with WADs have persistent pain and disability [7]. However, there are still conflicting views on etiology and pathophysiology [8], and so, whiplash injury remains a controversial subject, ranging from its definition to complex medical and legal issues [9,10].

### Web-Based Patient Information

Patient information is critical to the health care sector. If purposefully designed, this information can support patients in making well-founded decisions about their care, thereby facilitating patient-focused care. In Sweden, patient information is mainly found at the Healthcare Guide 1177, a national portal for health care advice and information [11]. The guide’s explicit aim is to increase access to health care, strengthen the patient’s role, and improve public health by offering the general public both in-depth information and access to the health care system. The content is quality assured in collaboration with experts. Access to information can be gained via the home page by clicking the heading entitled *Facts & Advice*. This loads a Web page with 40 broader medical areas listed in alphabetical order. Locating and clicking on *Joints, Muscles & Bones* will load an alphabetical list of more detailed conditions where *Whiplash Injury* is included. More practically, the same information can always be accessed by using the portal’s search field found at the top of the home page. The information analyzed in this study includes sections for injury mechanisms, symptoms and diagnosis, and treatment and care (Multimedia Appendix 1), which are the main headings found in all condition-specific information at the portal.

It is not unusual for patients to be dissatisfied with the information they have been given because the information often fails to consider individual needs in a satisfactory way [12]. Whether patient information works well (ie, provides the material needed for good patient decision-making) is dependent on, among other things, inclusion of end users during the design process (Textbox 1) [13,14].

With highly debated issues like whiplash injuries [9,10], it is important to involve patients in the design of patient information, so that their ideas and needs can be incorporated. This way, the information compiled can be made more relevant to the target group [15].

### Study Aims

By focusing on respondents’ perceptions, this research aims to describe how patients and relatives perceive (1) information about whiplash injuries found on the the Swedish national Web-based portal for health care advice and information (version 2012-12-07-2016-06-21; Multimedia Appendix 1) and (2) the public health care system’s management of whiplash injuries. The goal was to contribute to the improvement of such patient information.

This study was initiated because of acknowledged shortcomings in the guide’s information about whiplash injuries—the editor of the Healthcare Guide 1177 had received several requests from different parties for a general update of the information and extended an invitation to the corresponding authors’ research group to provide the suggested update. The information was updated in collaboration with the corresponding author (2016-06-22) before this study was published.

**Variables influencing the quality of patient information according to Bunge et al [13]. Patients’ participation is included in the last variable development process.**

- Content of information and meta-information
- Quality of evidence
- Patient-oriented outcome measures
- Presentation of numerical data
- Verbal presentation of risks
- Diagrams, graphics, and charts
- Loss- and gain-framing
- Pictures and drawings
- Patient narratives
- Cultural aspects
- Layout
- Language
- Development process

## Methods

### Study Design

With the aim of describing how patients and relatives perceive Web information about and the health care system’s management of whiplash injuries, we set out to engage focus groups [16] for interviews and to assess the resulting data through qualitative content analysis. Content analysis allows for the interpretation of underlying meaning(s) by penetrating the respondents’ choices of phrases and words in relation to context [17].

### Recruitment

Respondents were purposely recruited with one distinct criterion for inclusion—anyone who was part of the target audience of the Healthcare Guide 1177. Other inclusion criteria included: (1) experienced a whiplash injury in the past, or is the relative of someone with a previous whiplash injury and (2) a willingness to share experiences and perceptions of the patient information found on the guide with a researcher. We chose to engage members of a patient organization with the aim to obtain rich and relevant data. Patient communities tend to have an ongoing exchange of experiences, reflection, and knowledge accumulation. This awareness of relevant issues in the field made the respondents better prepared to discuss the pros and cons of the patient information, compared with the average person or nonengaged individuals [18]. Study participants were contacted via four Swedish patient-driven organizations representing the interests of people with neck injuries (the Swedish Neck Injury Organization; the Rights of Persons With Neck, Back, and Brain Injuries to Assessment and Diagnosis After Trauma; the Swedish Association for Survivors of Accident and Injury; and the Trigger Point Association). The associations were asked to randomly gather names of patients and relatives. To a group of about 100 patients and relatives recruited by the associations, we sent an information letter and a consent form, through which 52 individuals agreed to participate by returning the signed consent form. An email invitation to a focus group interview was sent to 28 individuals from this group who could be reached by email. Finally, the respondents were those who were able to attend the interviews, until we estimated that saturation was reached.

The first interview served as a pilot. Two respondents participated and the interview was held using the same question template as in the subsequent interviews. We chose to include the first interview in our material as it worked well and produced answers relevant to the study objective. The matters of importance emerging in the pilot interview remained consistent in all 5 interviews. Already in the first couple of interviews, the participants’ perceptions converged on a limited number of matters, with new participants confirming and adding their own experiences on already existing matters. This, in our view, suggests that the number of respondents, however small, was nevertheless sufficient to produce adequate results.

### Study Participants

The 13 study participants, 9 women and 4 men, were aged between 35 and 74 years, and 10 of them had had a whiplash injury. Time elapsed since diagnosis varied between 6 to 28 years.

### Data Collection

Data were collected in the autumn of 2015 in Stockholm. A total of 5 interviews were held within a month. Semistructured interviews were used (key questions and discussion), one of which took the form of a focus group with several participants. Three interviews with groups of 2 (patients), 1 (relative), and 7 (patients) participants were held by telephone for geographical reasons and 2 interviews were held in physical meetings with 1 (patient) and 2 (relatives) participants. The physical meeting interviews were held at a primary health care center in Stockholm, Sweden, in a comfortable, simply decorated conference room. Respondents who came to a physical meeting interview were offered coffee or tea and sandwiches. There was no other compensation or reimbursement.

We used a simple question template (Multimedia Appendix 2) that corresponded to the headings in the existing patient information at the Healthcare Guide 1177 and asked broadly about perceptions of information on injury, perceptions of injury, and perceptions of care. Questions were phrased in a way that made it possible for the respondent to give his or her view on many different aspects of the text such as “How did you perceive the information at 1177.se?” At the start of each interview, respondents were asked to take about 20 min to reread the text for better recall. The interviews were 60 to 90 min long and were taped with a sound recording device. At the end of each interview, one of the researchers summarized what was discussed and asked if anything was missed. Each interview was transcribed and printed.

Three researchers held the interviews (2 present at each interview), 1 family physician (male), 1 psychotherapist and medical student (male), and 1 physiotherapist and linguist (female), the latter with many years of professional experience in sociolinguistics.

### Data Analysis

The interviews were followed by a step-by-step conventional content analysis, where all meaningful interview text units are extracted and condensed into codes, after which the codes are grouped into subthemes defined by Graneheim and Lundman [19] as “threads of meaning running through the condensed text.” Finally, the subthemes were abstracted into overall themes. The psychotherapist and physiotherapist coded all 5 interviews together, aligning differences in assessment. Latent content was labeled as subthemes and themes to capture the essence of the respondents’ descriptions [17]. The themes emerged toward the end of the analysis. The quotes were translated from Swedish to English by the first author and then reviewed by a native English proofreader.

### Ethical Considerations

This study was conducted in accordance with the Declaration of Helsinki [20]. Before commencing data collection, ethical permission was granted by the Ethical Review Board in Stockholm (2015/1629-31/5). The respondents’ privacy was guaranteed by confidentiality during the entire study. The patient organizations were informed both orally and by email about the study and its terms and purpose. Each organization provided the same information to potential participants individually. All the respondents gave their written consent after being informed of the study aim, of their rights, and that participation was voluntary in all aspects. Each interview began only after the moderator reminded the respondent or respondents of the recordings.

## Results

### Overview

The respondents are referred to as R1 to R13 and as *she* throughout the text below. No significant differences were found between patients and relatives as both groups contributed in a similar way to each of the codes and subthemes.

Overall, 4 subthemes were developed inductively from 11 codes (13 codes in all). Two main themes, confidence and trust in health care and disappointment with health care contacts, were identified from the subthemes (Textbox 2). A total of 3 codes were excluded from further analysis (Table 1) as they did not relate to the research question.

### Subthemes

#### 1. A Bridgeable Knowledge Gap in Swedish Health Care

Respondents overwhelmingly expressed a general lack of basic knowledge in health care about causes and mechanisms of WADs. Neither 1177.se nor health care guidelines offered adequate facts or recommendations for problem management in the respondents’ view. As a result, patients and the public were more ignorant than initially thought, considering the abundance of knowledge available today. The lack of adequate management and a diagnosis was evident to several respondents:

From what I know today, my feeling’s that the guide is lacking in several places....There’s nothing at all here—how to care for, about what can come from this type of injury!

R12, 45-year-old relative

For me...it’s been such a long stretch. In the beginning...there was like, no process to begin with to investigate whether I had a neck injury.

R11, 52-year-old patient

Important facts not conveyed by the health care system were that even mild force can cause injuries and that being less physically active right after the accident may be beneficial. Moreover, one respondent said that listing symptoms in a primary care consultation did not “ring any bells” and resulted only in the advice to take a paracetamol.

Some had concluded that there lacked awareness of this group of patients in the Swedish health care system, since adequate care existed only outside of it. One respondent described never being presented with a proper diagnosis, only “late symptoms after a motor vehicle collision,” which she found hurtful and signaling a lack of interest in finding where the injury was located. For another, it had taken a long time just to get the information that it was a neck injury and years to get it documented.

According to another respondent, the phrasing of content found on 1177.se threw suspicion on affected individuals. She had interpreted the main message of the information on the portal to be that, after an accident, an injured person may find it pleasant to remain idle at home, which was improbable in her view. Overall, several respondents had their symptoms assessed as being mainly psychological. One respondent suggested that it may not be plain to the eye as to what was wrong, such as with a broken leg, and one had contacted a psychiatrist to fend off the hypothesis from health care professionals that the health issues faced by her relative were related to mental illness:

And the only one who tried to explain to NN what causes her to have these different symptoms...was really Dr NN. Because the others mainly offered her...well...that she had psychological...

R13, 74-year-old relative

Conversational therapy was proposed to provide symptom relief. The respondents reflected that this advice was unhelpful since talking to someone could not heal an injury:

Instead...they started to talk about...how she should go to a place for people who were burned out. So now she would travel tens of kilometres back and forth every day. And she...“I can feel I’m not burned out, that’s not the problem”...

R13, 74-year-old relative

One respondent worried about the consequences of the public’s view since the picture painted by 1177.se was that whiplash injuries tended not to be serious.

Several respondents suggested ways to provide symptom relief or prevention. Suggested strategies for higher quality and safer care included adequate information, pain medication, nerve blocks for pain treatment, cervical spine bracing, paid sick leave for a sufficient period of time, referral to therapists specialized in treating neck distortion, and help on learning to accept physical limitations and adjusting activity levels accordingly.

#### 2. Lack of Patient Safety

Respondents found that patient health and well-being could have been at risk if they had used 1177.se as a guide, either because of lacking information or a tendency to downplay or trivialize symptoms and concerns associated with whiplash injury. Several respondents found that basic information had been omitted. This would have kept patients and relatives unprepared and was a burden for them:

It would be a good thing if the Healthcare Guide could list the many similar problems you get initially, so you’ll understand that if this happens to you it’s part of the picture and you’re not the only one.

R2, 57-year-old patient

I would believe it until I...couldn’t believe it anymore...and that’s dangerous!

R7, 44-year-old patient

**Main themes crystallized from the interviews.**

- Confidence and trust in the health care system
- Disappointment with health care contacts

**Table 1:** Overview of the content analysis results.

Examples of meaning units	Codes^a^	Subthemes
“I’ve had a wonderful doctor who’s supported and helped me, and who had a personal interest in this type of injury, but many people with these injuries get doctors who don’t care and don’t take these things seriously at all. In my experience, the knowledge and interest of physicians out at the primary care centres varies and is a bit of a game of chance.” [R2, 57-year-old patient]	There are knowledge gaps	A bridgeable knowledge gap in the Swedish health care system
“I was given advice from two people who declared me an idiot and...gave me a psychiatric diagnosis on the phone! Because I didn’t have anything broken in my neck!” [R7, 44-year-old patient]	Psychologizing from health care	
“I so reject this argument that I expected to feel worse. What, why would I? I mean, I expected to feel better for a year. That was always my hope, that “this will get better, this will get better.”...In the end, you might accept it, but it’s not because of that that you get chronically ill, that you expected it.” [R3, 48-year-old patient]	My experience is questioned	
“Well, I’ve gone through about all of these treatments. But I think it’s important to get medication, to reduce the pain. As soon as possible, that is.” [R3, 48-year-old patient]	Examples of purposeful care or self-care	
“It was very hard to get any help. Our daughter sought help at the primary health care centre. But there was nothing wrong with her. They took X-rays and there was nothing wrong—that is, according to the doctors. But it turned out, since she’s been to NN, where we did this upright MRI, she has a lot of injuries. [...] Regular health care, there’s not a lot of help to get.” [R13, 74-year-old relative]	Develop adequate guidelines for investigation!	
“It says you should seek care at once if you have a lot of neck pain or headache after an accident. But I think some people haven’t had a lot of pain in the beginning, but the symptoms come later on!” [R4, 49-year-old relative]	Patient information is lacking	Lack of patient safety
“Let’s see what else I wrote. Vertigo, here, that vertigo can also be from anxiety. It doesn’t have to be. There may be injuries. There are actually quite delicate structures up there.” [R3, 48-year-old patient]	Downplaying from the health care system	
“How they describe that there are nerve fibres that transmit information...you really understand that there are things that can be injured. I find that this has improved!” [R3, 48-year-old patient]	Patient information is sufficient	The health care system as an authority
“The ones who must be informed are relatives and those in the periphery. Personnel at the social insurance agency, employers, colleagues, neighbours!” [R1, 35-year-old patient]	Patient information has an important role	
“And she has been active, as much as she could, given the injuries. So she didn’t...She’s really pretty tough. She’s been training and has tried to be active. She has dogs so she’s out a lot in the woods and fields. But the sort of help that you got at that pain unit...There’s no difference if you’ve got neck pain or shoulder pain or knee pain. Everyone had to do the same thing. So...they considered her as...like she wasn’t interested! When she couldn’t manage!” [R13, 74-year-old relative]	Feelings of abandonment	Lack of help and support
“These...interest groups, there’s really an immense amount of knowledge there.” [R3, 48-year-old patient]	Peer support is valuable	

^a^Three codes were excluded from the analysis as they did not relate to the research question: how I/my relative was injured, my/my relative’s symptoms, and my economy.

All respondents felt negative effects to the neck from a whiplash injury were downplayed in the patient information or by the health care system. The message in the patient information was that nearly everyone recovered, which did not correlate with the reality that many patients suffer from chronic symptoms. Health care contacts had said no injury could occur from a whiplash accident, although many experienced this. One respondent interpreted this to mean that health care personnel had no desire to investigate what was really wrong. Another respondent saw risk in the message to be physically active as soon as possible, as this may aggravate symptoms:

I’m thinking if there would be a ligament tear, this would need some rest to get a chance to heal.

R10, 52-year-old patient

The view was that there was a lack of expert knowledge and seriousness in the information available to patients, which meant people may seek care outside the public health care system. When to seek care after a whiplash injury was discussed. The patient information advised that if the symptoms did not decrease, or if there was substantial neck pain, to consider seeing a physician. Otherwise, patients were instructed to wait 2 weeks. This, it was pointed out, could create insurance difficulties for the individual because insurance agencies require that an injury be addressed within a few days or 72 hours. Respondents felt the patient information should advise a professional medical assessment at the slightest suspicion of injury.

#### 3. The Health Care System as an Authority

Several respondents shared views of medical and health care in terms of competence and reliability. The Healthcare Guide could be relied on; therefore, it was an important tool for obtaining information. One respondent allegorically described the significance of the Healthcare Guide as a bible. If someone’s child was sick, she said, there are always things you need to look up and then you turn to 1177.se. Also, the respondents felt Web-based patient information had an important role as an educational forum for the public. This was said to be even more important than informing patients and their families as it contributed to a general awareness in society. Several found positive aspects to the content of the patient information (eg, the possible symptoms of a neck distortion were well described). They found the version evaluated in the study to be better than previous ones and more comprehensive:

I rely on the Healthcare Guide as an instrument, so it’s actually quite important...

R1, 35-year-old patient

Had I gotten injured now today, and had turned to reading 1177, I’d have told myself...“this is going to be OK. I’m going to get well and I don’t have to dig into this. I don’t have to seek healthcare. This’ll pass!”

R7, 44-year-old patient

The respondents implied a trust in the public health care system and seemed to lack a critical view on the adequateness of instructions. For example, one respondent was treated in 20 sessions with no sign of improvement; however, she continued another 10 or 15 sessions. Later, she learned one might respond to some treatments and not others. Another respondent had felt pressured by the health care system to work more than her health allowed. Though it had felt wrong and better to care for her body, she granted the health care system an expert role. In this context, advice was followed regardless of outcome; the health care system seemed to hold an absolute mandate for assessment:

I was given the advice not to wear a cervical collar—and really I thought it was so stupid. I, like, walked around and—aaaah—instead of maybe being able to hold it together for a while.

R3, 48-year-old patient

Live life as normal, I was told. And you do follow what the doctor and the physiotherapist say to the dot.

R10, 52-year-old patient

#### 4. Lack of Help and Support

Several respondents perceived a lack of support from health care contacts. One respondent described feeling exposed and terribly alone following the accident; another respondent reported that next to no one had acknowledged that deteriorating mechanical properties could be a consequence of a neck sprain:

Um, and a lot of things like that, that makes me react when I see the text, a lot of glossing over saying it’s psychological, that the problems you have are psychological.

R3, 48-year-old patient

Yes, it’s called FMRI. I’ve had 2 independent assessments showing the exact same thing. But this wasn’t really taken seriously by the doctor and so on. It’s said it’s not scientific!

R8, 68-year-old patient

Moreover, it was said that 1177.se addressed individuals with other types of discomfort. Chronic problems were mentioned, but the text left one respondent with the impression that the intended target group did not include people in her situation. Help and support from peers rather than professional carers was described by several to have been helpful and empowering. Explanation and advice from peers helped respondents get oriented in the health care system, learn about treatment or know what questions to raise. All respondents described a struggle to find effective treatment and a more manageable situation for themselves or their relatives.

You can do stuff only you get so sensitive to strain, if you don’t have any recuperation time. This is the most misunderstood of all things. Namely that...not that you can’t walk, can’t lift, can’t laugh or whatever it might be. You can do everything. Only you’re so sensitive to strain.

R6, 53-year-old patient

So she was admitted to hospital. And no one wanted to look into it. She was hidden at a medicine ward. The kidney unit! Where all sorts of doctors were consulted. But no one wanted to deal with it. Instead...We chose ourselves or we decided together. That there won’t be any care. It’ll only get worse, and...And then I took her home in the same state as when she came in. She rode an ambulance back in full cramps again.

R12, 45-year-old relative

### Themes

Two main themes crystallized from the interviews, *confidence and trust in the health care system* and *disappointment with health care contacts* (Textbox 2). The main themes characterize the conflicting views on the patient information; the respondents’ needs and expectations and their experiences were in poor agreement with what the health care system had offered and the context of whiplash injuries described at 1177.se. In several cases, respondents found the facts (eg, about the type and severity of the condition) presented by the health care system to be misleading.

#### 1. Confidence and Trust in the Health Care System

The examples of cognition and behavior from the respondents revealed the long-term confidence and trust that characterized their relationship with the health care system. The respondents indicated an active participation in their care and no fear after the accident that they may be misled by the carer. They had felt secure and put themselves in the hands of the carer or health care as an institution, as one does in a trustful relationship. Important components of trust in a health care consultation are expectation and beliefs about the context, attributes of professionals, and the asymmetry of power between the carer and patient even if the relationship is voluntary.

All respondents, following the accident, acknowledged the health care system’s responsibility in providing recommendations. The patient information was seen as valid. Many facts had been excluded or omitted, which became clear later as respondents gradually gathered knowledge of the subject. They had not questioned the initial view of the situation, but felt surprised, perplexed, or misled when faced with the discrepancies between the briefing from health care encounters and the result of their rehabilitation. This was most likely a consequence of the general high regard for and trust in the competence of the health care system. In parallel, the recommendations available on 1177.se as to when one should seek care were vague, according to several people, and this instigated concern. It risked leaving to the patient or relative to decide what management action to take. It would have had negative consequences for the individual who sought advice if this advice had been acted upon. These opinions can be interpreted as requests for the health care system to assume greater responsibility, when phrasing definitions and in decision-making. It follows that respondents see the health care system as an agent qualified to set standards in the field. It also indicated that the role of the health care system strongly influenced the individual’s decisions:

There’s a risk among those who are injured, they remain at home waiting for 2 weeks...because it says so on the Healthcare Guide.

R2, 57-year-old patient

#### 2. Disappointment With Health Care Contacts

Respondents reported that their care had been inadequate in several ways and that very little or nothing had been done by the health care system to address it. Care management was below the standards they had expected, so they were disappointed—in the patient information description of whiplash as a minor injury, in their meetings with health care personnel, and in treatment outcomes. They felt let down in their situations and by the health care system. Given the seriousness of their problems, the likelihood that these individuals had set up *too narrow* parameters of an acceptable outcome was small. Most likely, they would have been content with partly helpful management; however, they had been deeply disappointed.

Several respondents described poor or inadequate management by the health care system compared to the management of other conditions. These descriptions ranged from risk-filled advice at 1177.se to wait and not obtain a medical assessment immediately following the accident to a lack of further investigation or referral to a more knowledgeable professional. The time span reflected this in several cases: sometimes many years passed until symptoms were traced to a neck injury or it was documented by the health care system:

Neurologists know surprisingly little. They have no interest in trying to solve the puzzle of what it’s all about. Orthopaedists have no interest either. It’s as if neck injuries didn’t exist in this country. That’s my experience.

R12, 45-year-old relative

It has taken me 15 to 20 years...I did not receive this information, not from the Healthcare Guide nor the Swedish healthcare.

R2, 57-year-old patient

We found that respondents perceived a breach of contract on the part of the health care system. First, they felt that the health care system had unloaded the responsibility of early management following a whiplash accident to the patient or relative. Second, health care had frequently failed to provide a basic level of help that is expected from a carer, summed in the often-cited Hippocratic oath to *console always, relieve often, and cure occasionally*. Instead, patients’ and relatives’ problems were ignored or bandied back at them:

No one here in Sweden ever acknowledged or cared for that.

R6, 53-year-old relative

## Discussion

### Principal Findings

The following 4 subthemes arose from the data: a bridgeable knowledge gap in the Swedish health care system, lack of patient safety, the health care system as an authority, and lack of help and support. A total of 2 main themes were identified that were significant for the respondents’ perceptions: confidence and trust in the health care system and disappointment with health care contacts. The themes revealed a gap between respondents’ needs and expectations and what the health care system had offered.

### Comparison With Previous Work

The majority of respondents reported that the type and severity of whiplash-associated disorders is trivialized at 1177.se. Respondents’ negative perceptions are fueled, for example, by the general psychologizing of discomfort. Although this is essentially a consequence of knowledge gaps and the omission of relevant facts in the information made available to patients via the health care portal, we suggest that the simplified presentation may have relevance. Linguistically, text can build group identity in the way content is selected and presented and the group is addressed and consequently handled. A communication strategy may make one group invisible by not mentioning it or refer certain behavior and values to another [21], as when respondents felt they were considered idle by the health care system. A possible influential factor may have been the Healthcare Guide’s editorial choices for a superficial structure (eg, syntax and word choice). Swedish county councils strive continually to write plainly [22], tailoring language for recipients, with the aim to support patients’ equal rights to participate in their own care. Patient information is recommended to suit the reading skills of 12-year-olds [13,23,24]. Such negative spillover from oversimplified presentation could be lessened by including in the patient information that the text is limited, general, and not for everyone (Textbox 3).

**Insights gained from the process of performing and analyzing the interviews and recommendations for those involved in designing patient information.**

- Use comprehensive and detailed information, as this can be very helpful in a serious health situation
- Include a statement that the text is limited, general, and not for everyone to avoid an element of exclusion of some readers or the impression that matters discussed are trivialized
- Include the target group(s) in the process of designing information to make the content relevant to readers

In this study, the perceived rejection from the side of the health care system caused great distress, as manifested in the respondents’ comments. When seeking help, the individual always exposes themself and their vulnerabilities to some extent to the carer or to the health care system. This demonstration of trust is most often accompanied by the expectation that others will respond to this openness in a responsible way. This has been described by Logstrup [25] as an *ethical demand*, which can be met or refused. For example, respondents clearly want more facts or all the available medical facts, especially of mechanisms behind the symptoms. The perceived lack of information caused misconception and worry. It is relevant to highlight earlier research focused on carer-patient communication for chronic conditions, where attempting to meet the patient in patient information has been discussed in terms of honesty. For example, among patients with cancer, honesty from carers is seen as helpful in making a serious health situation more real [26]. In another study on information for patients with cancer, the authors found that detailed information about a bad cancer prognosis does not cause more distress or diminish quality of life [27]. This indicates that even information conveying serious health issues can, in a complete and clear presentation of known facts, be helpful to the patient or relative. However, patient satisfaction with Web-based information in computer-based environments is, as of yet, not extensively researched. Brown et al [28] found that the overall desired elements of an electronic communication portal were frequent updates and detailed medical information rather than a presentation of the *big picture*. This was among American current and potential intensive care unit (ICU) patients and their family members. Rather than designing patient information by outlining matters of concern, the results in this study support the recommendation to use information-rich texts.

Brown et al [28] also found that preferences varied significantly by age, sex, ethnicity, and previous experience with ICU hospitalization. As for the management of chronic disease, Kruse et al [29] explored in a systematic review the shared characteristics of portals that receive favorable responses from patients and providers and elements that patients and providers believe need improvement. The authors concluded that there are varied attitudes among patients and caregivers toward the use of patient portals. The most prevalent positive attribute was found to be carer-patient communication, and the most prevalent negative perceptions were security and user-friendliness. Our results were homogeneous with regard to what patients expressed versus what relatives expressed. This must, however, be interpreted with care, as earlier research has found differences in patients’ and family members’ information needs [30].

Patient organizations are found by respondents to be valuable alternative sources of information. It is well known that copatients who have the same health issues can have a positive influence on patient empowerment, by providing skills, knowledge, and self-awareness [31]. Copatient narratives have also been shown to affect patient decisions on treatment [13], which can be beneficial or counterproductive to the patient. Both perspectives indicate a need to include the patient collective in a professional context if health care is to gain from peer support in treatment outcomes.

### Limitations

All the individuals participating in the interviews are members of a patient organization, and it may be that nonmembers with a whiplash injury in the past and their relatives face problems and challenges that members do not. In addition, 2 of the respondents worked in health care, which means that they can describe the patient or relative perspective with a greater understanding of the health care system. This may negatively impact the transferability of results. Finally, our study addresses the Swedish public health care context. However, though the results cannot be generalized to everyone experiencing whiplash, they can be of use in developing patient information, given the similarity of responses. There are frequently appearing common denominators. Another limitation is the difference in the number of participants between sessions. Group interaction is likely to have been more pronounced in the interview with 7 respondents than in the others. However, apart from the number of respondents, the interview format was the same and the nature of data collected did not differ across interviews.

Strengths of this work include the close relationship between participants’ descriptions that strengthens data credibility, the diversity of perspectives among the researchers, and coding results from consensus, which improves distinctness and subsequently the quality of the analysis.

### Conclusions

We found that most of the study participants felt distress due to insufficient information; respondents perceived a discrepancy between the public health care system's authority and the information provided. The Web information on whiplash injuries may greatly impact patients' care decisions as well as their physical, mental, and social well-being. We would recommend detailed patient information on whiplash injuries, with less emphasis on psychology and more data on pathophysiology, prognosis, and treatment.

## Appendix

Multimedia Appendix 1 Patient information from the Healthcare Guide 1177.

Multimedia Appendix 2 Interview question template.

## Abbreviations

ICU: intensive care unit
WADs: whiplash-associated disorders
