# Supplementary material for: “As If Neck Injuries Did Not Exist”: An Interview Study of Patients’ and Relatives’ Perceptions of Web Information on and Management of Whiplash Injuries in Sweden
Source: Interact J Med Res. 2019 May 21;8(2):e9881. doi: 10.2196/ijmr.9881 (PMC6547766; doi:10.2196/ijmr.9881)
Supplement: Multimedia Appendix 1 [file ijmr_v8i2e9881_app1.pdf]

Appendix 1 Whiplash Injury, version 2012-12-17–2016-06-21. Patient information from the Healthcare Guide 1177

Pisksnärtsskada – whiplashskada

Summary [Sammanfattning]

Allmänt

Man kan få en pisksnärtsskada om man till exempel blir påkörd bakifrån i trafiken och huvudet slungas bakåt och sedan framåt. Den hastiga rörelsen kan göra att muskler och vävnader i nacken och halsen påverkas.

Närmare hälften som får en pisksnärtskada blir bra igen efter ett par dagar och de allra flesta känner sig helt friska eller har bara lindriga besvär efter någon månad. Om besvären finns kvar efter ett år kan de bli mycket långvariga om det inte går att finna någon skada som kan behandlas.

Symtom

Symtomen efter en pisksnärtsskada visar sig ofta inom ett par dygn. Man kan till exempel

- ha ont och känna sig stel i nacken
- ha ont i bakhuvudet, armarna, axlarna och mellan skulderbladen
- känna pirrningar, stickningar eller domningar i armarna
- känna sig yr
- ha ont i ländryggen.

Om man får långvariga besvär kan man exempelvis få sömn- och koncentrationssvårigheter, sämre minne, huvudvärk och ångest.

Behandling

Om man fått en pisksnärtsskada bör man komma igång och röra sig så snart som möjligt, till exempel genom promenader och lättare nackrörelser.

Det finns olika sätt att behandla smärtan i nacken och halsen, till exempel genom receptfria smärtlindrande läkemedel, tens (transkutan elektrisk nervstimulering), akupunktur och sjukgymnastik.

Om man får långvariga besvär är det viktigt att man får det stöd man behöver för att hantera smärtan och andra besvär. Till exempel kan en allmänläkare eller smärtläkare, arbetsterapeut eller kognitiv beteendeterapeut vara till hjälp.

När ska man söka vård?

Om man är orolig när man har fått ont i nacken efter till exempel en bilkollision, kan man ringa sjukvårdsrådgivningen eller kontakta en vårdcentral.

Man ska söka vård direkt på en vårdcentral eller akutmottagning om man efter en olycka

- har mycket ont i nacken eller i huvudet
- känner en domnande och stickande känsla eller smärta i armarna.

Man kan alltid ringa sjukvårdsrådgivningen för att få råd.

What Happens in the Body [Vad händer i kroppen?]

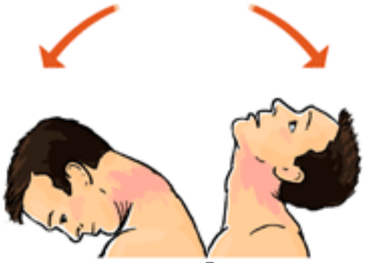

Huvudets rörelse liknar en pisksnärt.

Mer information

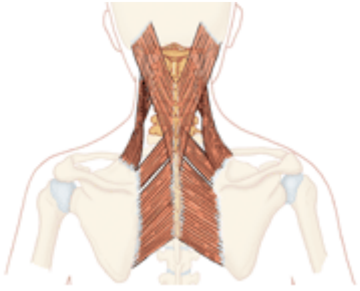

I halsryggraden ingår bland annat sju kotor som hålls på plats av kraftiga ledband och muskler.

Mer information

Troligen många orsaker till pisksnärtsskada

Ordet pisksnärt kommer från engelskans whiplash och beskriver den rörelse som huvudet och nacken kan utsättas för när man till exempel blir påkörd bakifrån i trafiken.

En pisksnärtsskada kan ge flera olika symtom. Det vanligaste är att man får ont i nacken och känner sig stel, men man kan också få huvudvärk eller yrsel, eller känna domningar i armarna. Ibland kan man även få ont i bröstryggen eller ländryggen.

Det är oklart vad en pisksnärtsskada beror på, men troligtvis är det många faktorer som påverkar. De flesta som får en pisksnärtsskada brukar bli bra inom någon månad, men en del kan få svåra besvär under lång tid.

Vanligast vid bilkollisioner

Den vanligaste orsaken till en pisksnärtsskada är en bilkollision. Skadan är lika vanlig när man krockar framifrån som när man blir påkörd bakifrån. Huvudet utsätts då för en rörelse som liknar en pisksnärt, och eftersom denna hastiga rörelse ökar huvudets tyngd orkar halsen och nackens muskler inte hålla emot. Det är denna rörelse som leder till att man skadas. Man kan också få en pisksnärtsskada när man utövar en del idrotter.

Nackens kotor, ledband och muskler

Med nacken menas lederna mellan huvudet och halsryggraden. Halsryggraden består av sju kotor som hålls på plats av kraftiga ledband och muskler. Kotorna förbinds med varandra dels genom mellankotskivor som sitter framtill, så kallade diskar, och dels genom facettlederna som sitter baktill på vardera sida av kotorna.

Vid varje rörelse man gör med nacken samarbetar olika halskotor, ledband och muskler. När man till exempel böjer huvud framåt, bakåt eller åt sidan rör sig alla sju halskotor.

Från halsryggraden går åtta par nervrötter till armens nerver. Varje nerv innehåller nervtrådar som leder information från hjärnan och ryggmärgen ut till kroppen, och nervtrådar som leder information från kroppen till hjärnan.

Kan bero på sträckningar i nackens vävnader

Orsaken till att man får ont eller känner sig stel i nacken efter en pisksnärtsrörelse kan vara att vävnader eller muskler i nacken fått sig en kraftig sträckning. Nackens muskler och vävnader är ofta svaga och rörliga och kan därför skadas när huvudets tyngd ökar under pisksnärtsrörelsen.

Den här typen av skador syns sällan utanpå eller med hjälp av vanlig röntgen. De brukar oftast läka av sig själva inom några veckor.

Olyckan i sig påverkar

När man varit med om en olycka som lett till en pisksnärtsskada kan man känna sig orolig och stressad. Man kan känna rädsla och ångest när man minns olyckan eller få sömnproblem och koncentrationssvårigheter. När man har ont är det dessutom lätt att man spänner sig, vilket kan leda till att man får fler besvär. Om smärtan blir långvarig och den påverkar sömnen långsiktigt, kan man få svårigheter att koncentrera sig.

Besvären kan ge andra symtom

När man har ont och är stel i nacken kan man också få huvudvärk. Smärtan kan ibland sprida sig till bakhuvudet, armarna, axlarna och käkarna. Hos vissa kan smärtan i käkarna leda till att man får svårt att gapa. Spänningar kan ibland leda till att man känner stickningar, pirrningar och domningar i armarna.

En del kan få sämre hörsel eller få tinnitus när de har ont i nacken och känner sig oroliga och rädda. Tinnitus är ljud som man hör utan att det finns en ljudkälla i omgivningen.

En pisksnärtsskada kan ibland leda till att man får yrsel. Yrseln kan ha flera orsaker. Den vanligaste är att man känner sig orolig eller chockad om man till exempel varit med om en olycka och därför spänner musklerna i nacken. Yrsel brukar för de flesta gå över av sig själv när man börjar röra på nacken.

Hur vanligt är det?

Det är svårt att veta hur många som får pisksnärtsskador, men forskning har visat att ungefär två personer per 1 000 i befolkningen skadas årligen i den svenska trafiken, eller cirka fem personer per 100 kollisioner med motorfordon.

Kvinnor tenderar att ha en något högre risk för att skadas, men det finns inga tydliga könsskillnader när det gäller risken för långvariga besvär.

Oklart varför vissa får långvariga besvär

Forskarna är ännu inte helt säkra på varför en del personer får långvariga besvär efter en pisksnärtsskada. Men det har visat sig att risken för besvär är större om man har mycket ont direkt efter skadan. Oftast spelar även andra orsaker in. Det kan till exempel vara svårare att återhämta sig om man redan tidigare haft besvär med smärta eller problem med nacken. Detsamma gäller om man är orolig och rädd för att röra sig efter olyckan.

Om man har ont under en längre tid kan smärtan göra att man blir känsligare för smärta eftersom nervsystemet påverkats. Då kan man ha ont även efter att den egentliga skadan har läkt. Att ha ont under en längre tid kan skapa oro för framtiden och göra att man till exempel blir nedstämd, får sömnproblem eller blir deprimerad.

Det har visat sig att risken för att få långvariga besvär ökar om man förväntar sig det. Därför är det viktigt att veta att de allra flesta blir bra efter en pisksnärtsskada.

Hur kan man förebygga långvariga besvär?

Långvariga besvär från en pisksnärtsskada kan förebyggas på flera olika sätt. Det är viktigt att man kommer igång och rör sig som vanligt så snart som möjligt efter skadan, eftersom besvären annars kan bli värre.

Numera finns det bilstolar och nackstöd som anpassar sig till kroppens rörelser i själva krockögonblicket vid påkörning bakifrån. Men det är ännu oklart om de kan minska risken för att man har besvär lång tid efter en

olycka. Om man ser i backspegeln att man är på väg att bli påkörd kan man försöka pressa ryggen och huvudet mot rygg- och nackstödet för att undvika pisksnärtsrörelsen. Man ska tänka på att ha nackstödet så högt och nära huvudet som möjligt när man kör bil.

Om man har varit med om en olycka är det normalt att man blir chockad efteråt. För att bearbeta chocken och undvika att besvären förvärras, kan det vara bra att tala med andra om vad som hänt. Det kan också vara till hjälp att undvika stress och ställa mindre krav på sig själv än vanligt. Att promenera och göra avslappningsövningar kan också hjälpa.

Hur kommer det att gå?

Ungefär hälften av dem som får en pisksnärtsskada blir bra redan efter ett par dagar, och de allra flesta blir helt friska eller har bara kvar lindriga besvär efter en månad.

Om man inte blir bra inom sex till tolv månader kallas besvären för långvariga. Då är det viktigt att man får det stöd och den hjälp som behövs för att hantera till exempel smärta och sömnsvårigheter. Man kan också känna oro och må sämre psykiskt när man haft ont under lång tid.

Ibland kan det vara svårt att klara även relativt enkla sysslor i vardagen, som till exempel att sköta tvätten, lyfta lätta föremål eller arbeta vid en dator. Även om man inte kan bli helt återställd kan symtomen ofta lindras och livssituationen förbättras med hjälp av till exempel mediciner, sjukgymnastik, avslappningsövningar, kognitiv beteendeterapi och anpassningar av arbetsplatsen.

Symptoms and Diagnosis [Symtom och diagnos]

Vanliga symtom på en pisksnärtsskada

Om man råkar ut för en pisksnärtsskada brukar symtomen ofta visa sig inom ett par dygn. Man kan

- ha ont och känna sig stel i nacken
- ha ont i huvudet eller mellan skulderbladen
- ha svårt att koncentrera sig, minnas saker eller sova
- känna sig yr
- känna smärta i bakhuvudet, nacken, armarna och axlarna som blir värre när man rör nacken
- känna pirrningar, stickningar eller domningar i armarna

Vissa kan också, även om det är ovanligt, få sämre känsel i en del områden av kroppen eller få svårt att gapa.

Långvariga besvär

Den smärta man känner i nacken kan ibland sprida sig till ryggen och armarna. Om smärtan övergår till kronisk värk kan det leda till att man blir mer känslig för smärta. Då kan man få värk i ländryggen.

Många som får långvariga besvär efter en pisksnärtsskada kan få svårare att sova, koncentrera sig eller minnas. En del kan bli deprimerade eller få ångest. Det kan bero på själva olyckan, men också på att man har ont och att livet har förändrats efter skadan. Vissa kan få problem med hörseln eller få tinnitus.

Man får berätta om olyckan

Om man söker vård får man ofta börja med att berätta om hur olyckan gick till. Läkaren får då en uppfattning av hur huvudet och nacken rört sig när olyckan inträffade, och vilken hastighet rörelsen haft.

Man brukar även få berätta var man har ont eller om man känner sig stel. Ibland får man själv försöka bedöma hur stark smärtan är utifrån olika skalor.

Rörligheten undersöks

Läkaren undersöker hur ont man har och hur rörlig nacken är genom att man till exempel får böja huvudet framåt eller åt sidan. Läkaren brukar också känna med händerna längs nacken och ryggen för att se om man är öm.

Det kan vara svårt för läkaren att bedöma hur rörlig man är om man spänner sig på grund av att det gör ont. Om man ligger ner kan man ibland känna sig mer avslappnad, och det kan underlätta en bedömning av rörligheten.

När man blir undersökt får man också ofta rådet att börja med lätta nackrörelser och annan motion så snart det är möjligt, till exempel regelbundna promenader. Syftet med aktiviteter är att skapa rörelse mellan kotorna utan att musklerna spänns och krampar.

Nerverna undersöks

Läkaren undersöker också hur nerverna fungerar genom att kontrollera känsel och reflexer. På så vis utesluts möjligheten att man har fått några nervskador vid olyckstillfället. Det är väldigt sällan som några nerver skadas vid pisksnärtskador. De domningar och pirrningar man kan få är ofta en typ av överförd smärta från nacken och ryggen och inte tecken på skadade eller klämda nerver.

Yrsel kan testas

Om man är yr kan man få gå igenom en undersökning där man först får lägga sig för att sedan ställa sig upp fort. Ibland kan man även få vrida huvudet från höger till vänster flera gånger. Undersökningarna leder ibland till att man känner sig yr. Men yrseln brukar gå över. Det kan vara svårt att gå igenom undersökningarna om man samtidigt spänner sig på grund av smärtan i nacken.

Svårt att gapa

Om man har svårt att gapa brukar man få försöka gapa och bita ihop så att läkaren kan se om man kan bita ihop helt och om bettet är snett. Svårigheter att gapa och bita ihop kan ibland bero på att musklerna i käken

är spända och man kan bli hjälpt av till exempel avslappningsövningar eller en så kallad bettskena.

Käklederna och nackens leder påverkar varandra, vilket gör att nackbesvär kan ge svårigheter att gapa och käkledsbesvär kan ge ökade besvär från nacken. Det är därför läkaren med undersökningen försöker skilja på vad som är vad. Om bettet är snett kan man få remiss till en tandläkare för bedömning. Ibland kan en bettskena göra att besvären minskar.

Viktigt att hantera reaktioner på olyckan

Läkaren brukar ställa frågor för att försöka få reda på om man visar några symtom på stress efter en olycka. Sådana symtom kan exempelvis vara sömnsvårigheter, ångest, koncentrations- och minnesvårigheter, eller tecken på depression. Det är viktigt att man berättar för läkaren hur man mår eftersom risken för långvariga besvär minskar om man tidigt får hjälp att hantera reaktioner på olyckan.

Röntgen och bildundersökning

Oftast behöver man inte röntgas eller gå igenom andra undersökningar. Men ibland kan läkaren vilja se om skelettet eller ledbanden har blivit skadade. För att utesluta skelettskador kan man då få nacken röntgad och för att utesluta diskbråck eller ledbandskador kan man bli undersökt med hjälp av en datortomograf eller en magnetkamera.

Datortomografi- och magnetkameraundersökningar ger mer detaljerade bilder än en vanlig röntgen, men de tar längre tid att genomföra.

Tre grader av pisksnärtsskada

Vilka undersökningar man får gå igenom beror till stor del på hur ont man har och hur svår pisksnärtsskadan bedöms vara.

Det finns en mall för att bedöma pisksnärtsskador som har arbetats fram av Whiplashkommissionens medicinska expertgrupp på uppdrag av Läkaresällskapet.

Pisksnärtsskador, eller WAD (Whiplash Associated Disorders), delas in i tre grupper beroende på vilka symtom man har:

- WAD-grad 1: Man har ont och känner sig stel i nacken. Oftast behöver man inte röntgas om man inte har någon annan sjukdom som kan påverka, till exempel någon reumatisk sjukdom.
- WAD-grad 2: Man har både ont och är stel i nacken, samtidigt som man har neurologiska symtom som till exempel smärta, domningar eller stickningar som strålar ut i armar, händer och fingrar. Tester tyder ändå på att man inte har några nervskador.
- WAD-grad 3: Utöver besvären i nacken och svårigheterna att röra sig har man kraftigare neurologiska symtom som till exempel att reflexerna inte fungerar som de ska, att man har svagare muskler eller sämre känsel. Om skadan klassas som WAD-grad 3 brukar man bli undersökt med en magnetkamera eller en datortomograf för att

läkaren ska kunna se om man har diskbråck, nervpåverkan eller ledbandsskador.

När man fått en diagnos
Om det känns skrämmande att få diagnosen pisksnärtsskada kan det vara bra att veta att de allra flesta blir bra igen. Diagnosen kan kännas oklar eftersom det handlar om en komplicerad skada som påverkas av olika faktorer. Det kan vara jobbigt att inte få en konkret förklaring till de besvär man har, eller att skadan inte går att behandla med en operation. Det är viktigt att man får information om vilka rörelser man kan göra för att bli bättre eller vilka smärtstillande läkemedel man kan använda. Man bör få komma tillbaka till läkaren inom en till tre veckor om de besvär man har behöver följas upp.

Om man behöver stöd
Om man får långvariga besvär innebär det förstås att livet förändras. När man har ont hela tiden kan både privatlivet och arbetslivet påverkas. Då är det viktigt att man får stöd och hjälp för att kunna hantera sorgen över att livet har förändrats och för att kunna förbättra sin egen situation. Sådan hjälp kan man till exempel få av en arbetsterapeut, sjukgymnast eller psykolog.

Care and Treatment [Vård och behandling]

När ska man söka vård?
Man bör söka vård direkt på en vårdcentral eller akutmottagning om man har mycket ont i nacken eller huvudet efter en olycka, eller känner en domnande och stickande känsla eller smärta i armarna. Om man har besvär med tinnitus eller yrsel efter en olycka och det inte går över efter ett par veckor bör man kontakta en vårdcentral.

Du kan alltid ringa och få sjukvårdsrådgivning på telefonnummer 1177.

Om besvären inte blir bättre
Om man efter två veckor fortfarande har ont i eller svårt att röra nacken, har återkommande huvudvärk, yrsel eller känner sig stressad och orolig bör man ta kontakt med en vårdcentral.

Ibland kan behandling hos sjukgymnast, kiropraktor eller naprapat skynda på läkningen om besvären inte försvinner inom en till två veckor. Det är viiktigt att man inte stelnar till och att man försöker undvika smärtsamma rörelser eftersom det kan öka risken för långvariga besvär. Om man stelnar till och har svårt att röra sig bör man därför söka hjälp för att komma igång med vanliga aktiviteter så snart som möjligt.

Vad kan man göra själv?
De flesta pisksnärtsskador läker av sig själva, men för att minska risken för långvariga besvär är det bra om man snabbt kan komma igång och röra sig

efter en skada. Man kan promenera och försöka göra lättare nackrörelser, men man bör undvika hårdare träning i början. Om det gör ont eller om man har svårt att röra nacken bör man vara försiktig med nackrörelser de första dagarna, men att försöka vila för att få bort värk i nacken kan på längre sikt förvärra smärtan. Det beror på att vävnaderna i nacken mår bättre av att vara i rörelse. Man ska inte använda nackkrage eftersom den kan öka besvären.

Lindra smärtan

De flesta som får en pisksnärtsskada behöver ingen behandling eftersom symtomen oftast går över av sig själva. Men om man har ont under den första tiden kan det vara bra att ta receptfria smärtstillande läkemedel som innehåller paracetamol, till exempel Alvedon och Panodil, eller antiinflammatoriska smärtstillande läkemedel, så kallade cox-hämmare, som Ipren och Naproxen.

Om man är över 75 år, har någon hjärt-kärlsjukdom eller tidigare har haft magsår bör man använda paracetamol istället för cox-hämmare.

Om de receptfria medicinerna inte hjälper kan läkaren även skriva ut andra smärtstillande mediciner. Det är viktigt att minska smärtan så att man kan komma igång och röra sig, och därmed ökar chansen att bli återställd.

Man kan få hjälp av en sjukgymnast att komma igång med bra rörelser och övningar som kan hjälpa mot stelhet. Sjukgymnasten kan också lära ut balansövningar om man har problem med yrsel.

Man kan behöva vara sjukskriven en tid om man har mycket ont i nacken eller har ett arbete som är olämpligt när man har nackbesvär.

Kyla eller värme kan hjälpa

Om man känner sig stel, öm eller har ont kan man pröva om det hjälper med kyla eller värme. Man kan till exempel lägga några isbitar i en påse och lägga den där det gör ont, eller hålla nacken och axlarna varma med en halsduk eller en värmedyna. Värmen kan göra att musklerna slappnar av.

Öva avspänning

Avslappning kan hjälpa mot både kortvariga och långvariga besvär och är oftast en mycket viktig del i läkningen. Under avslappningen kan man till exempel bli medveten om spänningar som uppkommit efter olyckan. Aktiviteter som skapar små rörelser mellan kotorna i nacke-rygg med bibehållen avslappad muskulatur är ofta bra. Det kan till exempel vara lugna promenader längs en stig i skog och mark, avslappad ridning i skritt eller klassisk skidåkning i långsamt tempo.

När man övar avslappning är samspelet mellan tankar, känslor, muskler och smärta viktigt. Det har även visat sig att de förväntningar man har på en förbättring har stor betydelse för att man ska bli bra igen.

Man kan bli hjälpt av en sjukgymnast om man har svårt att göra avslappningsövningar.

Andra besvär än smärta

Om man har problem att sova får man ofta svårare att hantera smärtan. Det kan därför vara viktigt att få hjälp med sömnen. Vanliga sömntabletter ger enbart stöd till insomning, vilket sällan är problemet efter en pisksnärtskada. I stället finns det andra mediciner som fungerar bättre genom att de höjer smärttröskeln och därigenom förbättrar sömnen.

Även avslappningsövningar och vissa typer av psykoterapi kan förbättra sömnen, till exempel kognitiv beteendeterapi som förkortas KBT. Med hjälp av KBT kan man lära sig strategier för hur man ska hantera till exempel sömnbesvär, smärta eller svåra situationer.

Om man blir orolig, får ångest eller blir deprimerad på grund av smärtorna och besvären kan även detta behöva behandlas med läkemedel, psykoterapi eller en kombination av både läkemedel och samtal. Det är viktigt att man tar upp sådana besvär med en läkare eftersom de gör att man har svårare att hantera situationen.

Om man får långvariga besvär

Har man fortfarande ont och svårt att arbeta eller klara av vardagliga sysslor efter en månad kan det vara bra att få skadan bedömd av mer än en person. Man kan till exempel behöva träffa en läkare som är specialist på smärta, eller en sjukgymnast, neurolog, ortoped, psykolog, socionom eller arbetsterapeut. När man lider av långvarig smärta bör man få en samlad utredning av hela sin livssituation, med till exempel fysiska, psykiska och sociala bedömningar, och kanske få en remiss till en smärtmottagning eller till en kurator.

Om besvären leder till att man inte kan arbeta under en längre tid måste man få hjälp med rehabilitering som syftar till att man ska kunna börja arbeta igen. Då ska arbetsgivaren samarbeta med sjukvården och Försäkringskassan. Det är viktigt att man hittar en metod som fungerar bra och kan leda till att man kommer tillbaka till arbetslivet.

Alternativa metoder kan hjälpa

Det finns flera alternativa metoder som kan användas när man haft ont under en längre tid. I dag används exempelvis akupunktur som smärtlindring av många sjukgymnaster. Metoden brukar användas som ett komplement till vanlig smärtlindring. När man får behandling med akupunktur används tunna, sterila nålar som sticks in på bestämda punkter på kroppen. Vilka punkter som behandlas beror på var man har ont. När man får behandlingen frigörs bland annat kroppens egna smärtstillande ämnen, så kallade endorfiner, som kan minska smärtan.

En annan metod som används för att minska smärta genom att frisätta endorfiner är transkutan elektrisk nervstimulering, så kallad tens. Med hjälp av en tens-apparat får utvalda muskler, senor och leder svaga elektriska impulser. Under behandlingen får man elektroder fästa på huden. Elektroderna är anslutna till en dosa som avger svaga elektriska impulser. Man upplever en surrande känsla i huden, som kan bli lite röd. Ibland kan man även få låna en tens-apparat för att kunna behandla sig själv hemma.

Att leva med en långvarig pisksnärtsskada

Om man har haft ont under en längre tid är det lätt att man oroar sig för att man aldrig kommer att bli bra igen. Man kan behöva bearbeta de känslor man har på grund av att livet förändrats sedan man börjat få ont. Man kan förlora kraft, ork, vilja, socialt liv, arbete och kanske även livsgnistan. Det kan också bli svårt att planera sitt liv från den ena dagen till nästa eftersom smärtan kan komma och gå och hindra att man gör sådant man vill och behöver göra.

Därför är det bra om man kan hitta flera olika sätt att minska smärtan och andra besvär. Det kan också hjälpa att försöka lära sig sätt att hantera smärtan och försöka få en så bra livskvalitet som möjligt.

Hur man mår spelar stor roll för förmågan att kunna hantera smärta, samtidigt som långvarig smärta också påverkar hur man mår psykiskt. Man kan bli hjälpt av att gå i någon form av samtal hos till exempel en kurator eller psykolog eller gå i särskilda smärtskolor.

Redaktör:

Peter Tuominen, 1177.se

Granskare:

Lena Holm, epidemiolog, Institutet för miljömedicin, Karolinska Institutet, Stockholm

Illustratör:

Lotta Persson, illustratör, Göteborg

Kari C Toverud, certifierad medicinsk illustratör, Oslo, Norge
