# Supplementary material for: “As If Neck Injuries Did Not Exist”: An Interview Study of Patients’ and Relatives’ Perceptions of Web Information on and Management of Whiplash Injuries in Sweden
Source: Interact J Med Res. 2019 May 21;8(2):e9881. doi: 10.2196/ijmr.9881 (PMC6547766; doi:10.2196/ijmr.9881)
Supplement: Multimedia Appendix 2 [file ijmr_v8i2e9881_app2.pdf]

Appendix 2 Interview Question Template

Introduction question: Tell us about where you have gotten information about whiplash injuries from.

- Do you remember the first time you sought information at 1177.se/consulted the Heathcare Guide? What was your thoughts about it? Why?
  - The text?
  - Anything in particular you reacted on?
  - Faults in the information?
- Now that you have read the text – What is your opinion? Why?
  - Easy/hard to read?
  - Informative or not?
  - Section for summary? Section for what happens in the body? Section for symptoms and diagnosis? etc.
- Something you want to add?
  - Something you want to include to contribute to better health care and better information?
- Any suggestions to us as researchers?

Summing up session by the observer, ended with: Do I perceive this correctly?

- What did you think of this meeting
